# Notes on Preparations for the Sick

**Published:** 1903-12

**Authors:** 


### Notes on Preparations for the Sick.

**Cheltine Nutrient Milk Chocolate, Cheltine Malted Milk Chocolate, Cheltine Soluble Maltose Food.**—CHELTINE FOODS, LIMITED, Cheltenham.

The Nutrient Chocolate contains nearly 30 per cent. of proteid food in combination with 27 per cent. of fat and nearly 42 per cent. of sugar constituents. It is a very acceptable sweetmeat, and should be a very convenient and nutritious kind of food for children and adults both in health and disease.

The Malted Milk Chocolate contains a proportion of the Soluble Maltose Food in addition to full cream milk. The combination has 51 per cent. of maltose and cocoa extractives, with 35·52 per cent. of fat and 12·50 per cent. of proteid. The preparation has a malty flavour, and its composition shows that it must have a high nutrient value.

The Soluble Maltose Food, for invalids and infants. This food is stated to contain no starch, and it requires no boiling; it should be mixed with a little hot water, to which milk may be added ; it does not require sugar.

The *Lancet* analysis is as follows :—

| | | |
|---|---|---|
| Moisture .. ... ... ... ... ... ... ... | 3·90 | per cent. |
| Malt and Milk-Sugar ... ... ... ... | 50·44 | ,, |
| Dextrin and other Carbohydrates ... | 34·06 | ,, |
| Proteid ... ... ... ... ... ... ... ... | 7·90 | ,, |
| Fat ... ... ... ... ... ... ... ... ... | 1·40 | ,, |
| Mineral matter ... ... ... ... ... ... | 2·30 | ,, |
| | 100·00 | |

The preparation by itself is, of course, deficient in fat, but the food is intended to be made with milk, and thus a product is obtained which contains an excellent proportion of all classes of nutritive material.

An examination of this food by Mr. Oliver C. M. Davis (University College Laboratory) shows that it is almost completely soluble in water, and that it does not contain starch. Carbohydrates are represented by maltose and dextrin, there being a high percentage of the former substance. A fair proportion of proteid matter is present, so that the food contains much nourishment in an easily assimilated form suitable for the infant and for the adult whose digestion is impaired.

The food is quite palatable, and is easily prepared inasmuch as it does not need to be boiled.

---

**Nestlé's Condensed Swiss Milk,** Prepared in Switzerland; **Nestlé's Viking Condensed Milk,** Prepared in Norway.—HENRI NESTLÉ, Christiania and Vevey. London depot: 48, Cannon Street.

The former has a small quantity of cane sugar added, the latter is prepared without the addition of sugar or any other substance. An analysis by Mr. Oliver C. M. Davis (University College Laboratory) shows the former to be a full cream condensed milk, with added cane sugar. The sample examined contained 12·5 per cent. lactose, 9·7 per cent. proteid, 9·8 per cent. fat, and approximately 35 per cent. of cane sugar. None of the nutrient matter of the milk has been removed in the process of condensation.

The Viking brand differs only from the above in that it appears to have no added sugar. None of the milk fat has been removed, and this preparation is recommended to those who are unable to take much cane sugar.

---

**Cocoa Essence, Mexican Chocolate, Milk Chocolate.**—CADBURY BROTHERS, Bournville.

These preparations are all excellent, as the name Cadbury is a sufficient guarantee. Chocolate and cocoa preparations are becoming increasingly popular, and need no commendation. They have a high value from the nutrient standpoint, and there is nothing to be said against their greatly extended use.

---

**Potter's Malt Egg.**—POTTER & CLARK, 60 Artillery Lane, London, E.C.

This preparation is an Extract of Malt prepared "in vacuo," with which is incorporated yolk of egg. The diastatic value of the malt has not been impaired during the manufacture, nor have the proteids of the egg been coagulated. An analysis (Mr. Oliver C. M. Davis, University College Laboratory) indicates the presence of albumins and globulins in large amount, also maltose, but no added sugar. There is only a slight amount of ash, which contains phosphates. The substance is very pleasant to the taste, and should be readily taken by children.

---

**Jeyes' Soluble Fluid. Creolin** (Disinfectant), **Creolin** (Medical).—JEYES' SANITARY COMPOUNDS, 64 Cannon Street, London.

These fluids have established for their promoters a well-deserved reputation. The soluble fluid when mixed with water gives a clear solution (not an emulsion): it consists largely of meta-cresol and para-cresol in a pure state. These bodies are kept in solution by means of a saponifying agent which appears to be a soda soap.

There are no indications of the presence of any metallic antiseptics.

The Medical Creolin is a purified form for internal administration and medical purposes generally. We are informed that an examination of this fluid (University College Laboratory) shows it to contain large amounts of the isomeric cresols, and in addition the higher homologues of the cresols appear to be present, inasmuch as substances with higher boiling.points than cresols were separated on distillation. Its value as a germicide has been abundantly established: it is found to be sixteen times stronger than carbolic acid, whilst its toxic and irritating properties are many times less. For surgical uses a strength of one in two hundred is sufficient.

---

**The Izal Vapouriser.**—NEWTON, CHAMBERS & Co., Thorncliffe, Sheffield.

This little apparatus consists simply of a brass cup from which the Izal disinfectant can readily be volatilised by means of an ordinary night-light.

Everyone is familiar with the powerful and reliable antiseptic action of Izal, even when diluted with one hundred parts of water. To its many surgical uses there is no need to allude. The vapouriser is especially intended for evaporating fluids in the patient's room in cases of croup, whooping cough, phthisis, and other affections where antiseptic inhalations are likely to be useful. Dr. Tunnicliffe reports much benefit in cases of phthisis, and Colin Campbell uses Izal for intra-tracheal injection.

---

**Arabella Natural Mineral Water.** — ARABELLA LIMITED, London, W.

This natural water, bottled at the springs, Kolenfeld, Budapest, has a specific gravity of 10.386 and contains 41.312 grammes per litre of mineral substances, of which about ninety per cent. consist of the sulphates of magnesium and sodium, with small quantities of the carbonates of magnesium and calcium.

Its composition gives a sufficient indication of its probable action, and experience shows that those who expect a definite aperient result will not be disappointed.

---

We have received samples of many of the **newer products** of Messrs. OPPENHEIMER, SON & Co., 179 Queen Victoria Street, London.

Many of these were exhibited at the Swansea meeting of the British Medical Association, and are familiar to most physicians. Some of the less familiar are worthy of mention.

**Aseptoids** are tablets which afford a ready and convenient means of instantly making a solution for nasal use or gargles.

**Cerettes** are neat flexible containers, made of gelatine and filled with ointments or medicated soaps, each cerette holding sufficient for one application.

**Cocoids** are tiny pastilles containing medicaments of fixed quantities carefully intermixed with a palatable chocolate basis.

**Rectones** are improved nutrient and medicated suppositories.

**Ergole** is a new preparation of Ergot possessing three times the strength of the B.P. preparation.

**Laxoin** is a synthetic laxative, which is palatable and does not gripe; its effect is not lost by repeated administration, and it may be taken without harm for long periods of time.

**Maglactis** (milk of magnesia) is a creamlike fluid containing magnesium hydrate, which is antacid and soothing in cases of gastric catarrh.

**Pinheroin** is a palatable combination containing terpin hydrate gr. j., heroin hydrochlor gr. $\frac{1}{40}$ in each fluid drachm, with essence of Canadian pine.

**Renaglandin**—the expressed extract of the suprarenal gland—is an aseptic concentrated extract, containing all the active principles in natural solution. It has proved to be a most efficient styptic. Prepared in the form of rectones, it is a very useful agent in the treatment of hemorrhoids.

The **improved Aërizer** is an instrument which renders fluids as fine as smoke, and thus enables medicated vapours to be inhaled into the smaller branches of the bronchial tubes, so directly reaching the affected lung tissue. This spray, with Neboline Compound, No. 21, has been found to be very useful in the treatment of asthma.

